# Hippocampal volume predicts antidepressant efficacy in depressed patients without incomplete hippocampal inversion

**DOI:** 10.1016/j.nicl.2016.04.009

**Published:** 2016-04-27

**Authors:** Romain Colle, Claire Cury, Marie Chupin, Eric Deflesselle, Patrick Hardy, Ghaidaa Nasser, Bruno Falissard, Denis Ducreux, Olivier Colliot, Emmanuelle Corruble

**Affiliations:** a INSERM UMRS 1178, Team “Depression and Antidepressants”, 94275 Le Kremlin Bicêtre, France; b Univ. Paris-Sud, Faculté de Médecine Paris Sud, 94275 Le Kremlin Bicêtre, France; c Service de Psychiatrie, Hôpital Bicêtre, Hôpitaux Universitaires Paris Sud, Assistance Publique-Hôpitaux de Paris, 94275 Le Kremlin Bicêtre, France; d INSERM U1127, F-75013 Paris, France; e CNRS, UMR 7225, 75013 Paris, France; f Sorbonne Universités, UPMC Univ Paris 06, UMRS 1127, F-75013 Paris, France; g Institut du Cerveau et de la Moelle épinière, ICM, F-75013 Paris, France; h Inria, Aramis project-team, Centre de Recherche de Paris, France; i AP-HP, Hôpital de la Pitié-Salpêtrière, Departments of Neuroradiology and Neurology, F-75013 Paris, France; j Université Paris-Saclay, Univ. Paris-Sud, UVSQ, CESP, INSERM, Villejuif, France; k CNRS IR4M, UMR 8081, 94275 Le Kremlin Bicêtre, France; l Service de Neuroradiologie, Hôpital Bicêtre, Hôpitaux Universitaires Paris Sud, Assistance Publique Hôpitaux de Paris, 94275 Le Kremlin Bicêtre, France

**Keywords:** HV, hippocampal volume, IHI, Incomplete Hippocampal Inversion, MDD, Major Depressive Disorder, MDE, Major Depressive Episode, Major Depressive Disorder, Hippocampal volume, Incomplete Hippocampal Inversion, Antidepressant, Response, Remission

## Abstract

**Background:**

Incomplete hippocampal inversion (IHI), also called malrotation, is a frequent atypical anatomical pattern of the hippocampus. Because of the crucial implication of the hippocampus in Major Depressive Disorder (MDD) and the neurodevelopmental hypothesis of MDD, we aimed to assess the prevalence of IHI in patients with MDD, the link of IHI with hippocampal volume (HV) and the impact of IHI on the predictive value of HV for response and remission after antidepressant treatment.

**Methods:**

IHI (right and left, partial and total and IHI scores) and HV were assessed in 60 patients with a current Major Depressive Episode (MDE) in a context of MDD and 60 matched controls. Patients were prospectively assessed at baseline and after one, three and six months of antidepressant treatment for response and remission.

**Results:**

The prevalence of IHI did not significantly differ between MDD patients (right = 23.3%; left = 38.3%) and controls (right = 16.7%; left = 33.3%). IHI was not significantly associated with MDD clinical characteristics. IHI alone did not predict response and remission after antidepressant treatment. However, an interaction between left HV and left IHI predicted six-month response (p = 0.04), HDRS score decrease (p = 0.02) and both three-month (p = 0.04) and six-month (p = 0.03) remission. A case-control design in 30 matched patients with or without left IHI confirmed that interaction. In patients without left IHI, left HV at baseline were smaller in six-month non-remitters as compared to remitters (2.2(± 0.43) cm^3^ vs 2.97(± 0.5) cm^3^ p = 0.02), and in six-month non-responders as compared to responders (2.18(± 0.42) cm^3^ vs 2.86(± 0.54) cm^3^, p = 0.03). In patients with left IHI, no association was found between left HV at baseline and antidepressant response and remission.

**Conclusion:**

IHI is not more frequent in MDD patients than in controls, is not associated with HV, but is a confounder that decreases the predictive value of hippocampal volume to predict response or remission after antidepressant treatment. IHI should be systematically assessed in future research studies assessing hippocampal volume in MDD.

## Highlights

• Incomplete hippocampal inversion (IHI) is not significantly more frequent in MDD than in controls.
• IHI is not significantly associated with MDD clinical characteristics.
• Hippocampal volume predicts antidepressant efficacy in MDD patients without IHI.
• Hippocampal volume does not predict antidepressant efficacy in patients with IHI.

## 1 Introduction

**Fig. 1:**
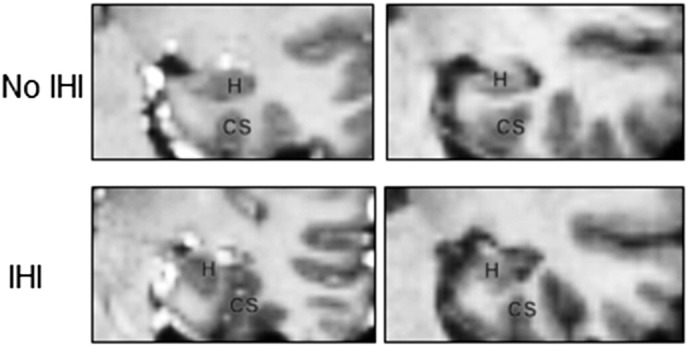
Example of temporal media lobe without and with incomplete hippocampai inversion in MRI coronal view. Top row shows hippocampl (H) without incomplete hippocampal inversion (IHI), they have a flat shape and horizontal orientation and normal sulci shape. Bottom row shows hippocampi (H) with IHI, they have a rounded shape and a medial positioning; the left one presents a deep and vertical collateral sulcus (CS).

Incomplete Hippocampal Inversion (IHI), also called hippocampal malrotation, is an atypical anatomical pattern of the hippocampus (Bajic et al., 2008, Baulac et al., 1998, Bernasconi et al., 2005, Cury et al., 2015). Its main features are a round and vertical hippocampal body in a coronal plane, a medial positioning of the hippocampus and a deep and vertical collateral sulcus (Bajic et al., 2008, Baulac et al., 1998, Bernasconi et al., 2005, Cury et al., 2015) (Fig. 1).

IHI was initially described in patients with epilepsy, with a prevalence of 30% to 50% (Bajic et al., 2009, Bajic et al., 2008, Baulac et al., 1998, Bernasconi et al., 2005, Lehericy et al., 1995, Raininko and Bajic, 2010). However, IHI is not specific to epilepsy and has also been reported in healthy subjects (Bajic et al., 2008, Bernasconi et al., 2005, Bronen and Cheung, 1991). A recent study assessed the prevalence of IHI in the general population in a large sample of over 2000 subjects (Cury et al., 2015). IHI can be total (all the criteria for IHI) or partial. It is a frequent phenomenon in healthy subjects and is more frequent in the left (17.1% for total IHI and 11.9% for partial IHI) than in the right hippocampus (6.5% for total IHI and 9% for partial IHI)(Cury et al., 2015). Although its frequency is lower than in patients with epilepsy, IHI is thus a common phenomenon in the general population and should be taken into account in morphometric studies. Furthermore, IHI is shown to impact the accuracy of automatic hippocampal segmentation methods and may thus be a cofounder of volumetric analyses (Kim et al., 2012).

IHI is thought to be of developmental origin, as shown by studies in neonates (Raininko and Bajic, 2010, Righini et al., 2006). Furthermore, IHI is associated with different developmental defects, including agenesis of the corpus callosum (Atlas et al., 1986), and genetic abnormalities (Andrade et al., 2013, Atlas et al., 1986, Baker et al., 2011, Boronat et al., 2015, Campbell et al., 2006, Grosso et al., 2003). Finally, subjects with IHI display morphological changes in several sulci outside the medial temporal lobe (Cury et al., 2015). These different elements suggest that IHI may thus be a sign of atypical brain development.

The hippocampus is a key cerebral structure for the physiopathology of Major Depressive Disorder (MDD), it is associated with memory function and a focus of neurogenesis (MacQueen and Frodl, 2011). Several studies have shown that MDD patients have smaller hippocampal volumes (HV) than controls (Kempton et al., 2011). Moreover, in MDD patients, smaller HV predict lower response and remission after antidepressant treatment, as shown in a recent meta-analysis (Colle et al., submitted for publication) of 6 studies, of which 2 were positive and 4 were negative (18–23). However, the effect size is moderate. Therefore, response and remission after antidepressant treatment are still difficult to predict in depressed patients (Kupfer et al., 2012). To take into account hippocampal atypical anatomical pattern in addition to HV could lead to improve accuracy of prediction of response and remission after antidepressant treatment.

Despite the major implication of the hippocampus in Major Depressive Disorder (MDD), the neurodevelopmental hypothesis of MDD that suggests that early developmental defect could contribute to the physiopathology of MDD (Ansorge et al., 2007), the impact of IHI on MDD has never been explored.

Thus, we aimed at answering the following five questions. Is IHI more frequent in MDD patients than in controls? Is IHI associated with specific clinical characteristics of MDD? Is IHI related to hippocampal volume in MDD? Is IHI a predictor of response/remission after antidepressant treatment in MDD patients? Does taking into account IHI improve the predictive value of hippocampal volume in the prediction of response/remission after antidepressant treatment?

## 2 Materials and methods

### 2.1 Design

Hippocampal volume and IHI were assessed at baseline in a 6-month prospective real-world setting treatment study of patients with a current Major Depressive Episode (MDE), clinically assessed at the beginning of antidepressant treatment, 1, 3 and 6 months later and in controls in a case control design. This study was registered by the French National Agency for Medicine and Health Products Safety (ANSM) and the Commission Nationale de l'Informatique et des Libertés (CNIL), was approved by the Ethics Committee of Paris-Boulogne, France, and conformed to international ethical standards. All patients provided written informed consent for study participation.

### 2.2 Patients and controls

60 consecutive in- or out-patients, aged 18 to 65 years, with a current Major Depressive Episode in a context of MDD, with a minimum score of 18 at the Hamilton Depression Rating Scale-17, HDRS-17 (Hamilton, 1960) were included. They had had a cerebral MRI in the Neuroradiology department of Bicêtre Hospital and were assessed for depression at the start of the index antidepressant treatment (M0), and one (M1), three (M3) and six (M6) months later.

Patients with psychotic symptoms, bipolar disorders, psychotic disorders, eating disorders, current substance abuse or dependence, pregnancy, organic brain syndromes or severe unstable medical conditions were not included. Patients receiving antipsychotics or mood stabilizers before inclusion and/or for four months or more during the last year were also excluded. Antipsychotics, mood stabilizers and stimulants were not permitted during the study. Benzodiazepines at the minimum effective dose and for the minimum duration and psychotherapies were allowed. The index antidepressant treatment had to be a monotherapy. The decision regarding the drug and its dose were left to the treating psychiatrist, using “real world” treatment options.

The index antidepressant treatment had to belong to one of the three following classes: Selective Serotonin Reuptake Inhibitors (SSRI) (n = 20), Serotonin Norepinephrin Reuptake Inhibitors (SNRI) (n = 34) and Tricyclic antidepressants (TCA) (n = 6).

60 controls who had benefited in clinical practice (mainly headache, migraine and vertigo exploration and search for cancer-associated brain metastases) from a cerebral MRI, in the Neuroradiology department of Bicêtre Hospital, for whose no neurological abnormalities were found on the MRI, and matched for sex, age, MRI acquisition type and MRI acquisition date with the 60 depressed patients were also included. Controls with psychiatric disorders, current substance abuse or dependence, as well as pregnancy, breast feeding, organic brain syndroms, dementia, epilepsy, or unstable medical conditions were not included.

### 2.3 MRI acquisition

Brain MRI acquisitions were performed on 1.5 (n = 92) or 3-T (n = 28) Philips systems. All subjects were scanned with a routine whole brain T1-weighted 3D sequence.

These images were acquired with a resolution of either 0.6 × 0.6 × 0.7 (interpolated) or 0.88 × 0.88 × 1.1 in sagittal plane, or with a resolution of 0.94 × 0.94 × 1.00 in axial plane. MRI acquisition sequences were associated neither with age, gender, IHI prevalence nor with antidepressant response and remission.

### 2.4 Assessment of IHI

IHI assessment was performed using the protocol defined by Cury et al. (Cury et al., 2015) (according to Cury et al., 2015). This protocol comprises a global criterion named C0 and five individual criteria (named C1 to C5). The five individual criteria assess: the verticality and roundness of the hippocampal body, the depth and verticality of the collateral sulcus, the medial positioning of the hippocampus, the thickness of the subiculum and the depth of the sulci of the fusiform gyrus. The five criteria are graded from 0 to 2. C0 evaluates the presence of IHI globally and is defined as follows: 0 is given if the hippocampus has a common aspect, 1 is given if the hippocampus does not have a common aspect but not a total IHI which corresponds to a partial IHI, a grade of 2 is given if the hippocampus has a total IHI. They were all assessed in the coronal plane and separately for the left and right hippocampi. For more details about these criteria, the reader is referred to Cury et al. (2015). These criteria have been demonstrated to have high intra-rater and inter-rater reproducibility (Cury et al., 2015).

In the analysis, IHI as defined by partial IHI (score 1 of individual criteria C0) or total IHI (score 2 of individual criteria C0) was chosen as the primary outcome. Total IHI, IHI score (sum of individual criteria C1 to C5) and IHI score ≥ 4 (optimal threshold for total IHI in Cury et al., 2015) were taken as secondary outcomes.

IHI was assessed blind to patient/control status, clinical and volumetric data. IHI were evaluated on the total sample by an expert (CC), who had previously evaluated about 1000 subjects for the presence of IHI. In order to assess inter-rater reliability, 60 MDD patients were evaluated by a second rater (RC) after three training sessions. Consistently with the results reported in the paper of Cury et al. (2015), we found a strong inter-rater reliability (Cohen's kappa = 0.80).

### 2.5 Hippocampal volumetry

The segmentation of the hippocampus was performed using the fully automatic SACHA software (Chupin et al., 2007, Chupin et al., 2009, Colliot et al., 2008). This approach segments the hippocampus based on competitive region-growing between hippocampus and amygdala. It includes prior knowledge on the location of the hippocampus and the amygdala derived from a probabilistic atlas and on the relative positions of this structure with respect to anatomical landmarks, which are automatically identified. All resulting segmentations were assessed for segmentation quality (from 0 for worst quality to 4 for perfect quality) by trained raters (R.C and M.C), blind to the study group (patients versus controls) and sociodemographic and clinical data. Only high quality segmentations (quality score ≥ 2) were included in the analysis: three patients and four controls were excluded from the volumetric analyses. Automated segmentation was preferred to manual segmentation first because manual assessment cannot be performed blind to IHI, second because automated segmentation has been validated as compared to manual segmentation in previous studies (Dill et al., 2015), and because it is faster, requires less specific anatomical expertise and does not suffer from high intra- and inter-rater variability. SACHA was previously validated in depressed patients (Bergouignan et al., 2009).

Total brain volume was determined with SPM5 to analyze hippocampal volume (mean, right and left) adjusted to total brain volume. Because brain volume, age and gender are known to be associated with hippocampal volume, non-matched analyses were adjusted on brain volume, age and gender.

No statistically significant difference for segmentation quality scores was shown in patients with right (partial + total) IHI as compared to patients without right (partial + total) IHI (m(sd) 2.4 (0.7) vs m(sd) 2.8(0.5), p = 0.24), and in patients with left (partial + total) IHI as compared to patients without left (partial + total) IHI (m(sd) 2.6(0.5) vs m(sd) 2.8 (0.4), p = 0.28).

### 2.6 Assessment of response and remission after antidepressant treatment

The HDRS scale (Hamilton, 1960) was rated in depressed patients by trained clinicians at baseline, one month, three months and six months after the beginning of antidepressant treatment. These raters were blind from MRI data. The percentage of remitters six months post-treatment was defined a-priori as the main outcome measure. A HDRS score of seven or less at follow-up defined remitters. Secondary outcomes were the percentage of decrease of the HDRS score between baseline and follow-up and the percentage of responders, response being defined by a decrease in the HDRS score of at least 50% from baseline to follow-up.

### 2.7 Statistical methods

First, bivariate analyses were performed using χ^2^ tests for categorical variables and Wilcoxon tests for continuous variables. Linear or logistic regressions were used to assess interaction effects between IHI and HV on antidepressant efficacy (HDRS improvement, response and remission).

A Receiver Operating Characteristic curve was built to determine the optimal HV threshold associated with remission after six months of antidepressant treatment in patients without left IHI. A sensitivity/specificity analysis was performed on left hippocampal volumes.

All tests were two-tailed. Significance level was defined as p < 0.05. All tests were performed with R 2.15.3. A power estimation was performed with G*power 3.1.

## 3 Results

### 3.1 Sample

The 60 MDD patients and the 60 matched controls had a mean (sd) age of 45.7 (12.8) years for patients and 45.4 (12.8) years for controls (p = 0.89) and 36 (60%) were women in each group (p = 1.0). The mean (sd) hippocampal volumes did not significantly differ between MDD patients (right: 2.55 cm^3^ (0.61), left: 2.51 cm^3^ (0.54)) and controls (right: 2.53 cm^3^ (0.51), left: 2.58 cm^3^ (0.47)).

### 3.2 Is the prevalence of IHI higher in MDD compared to controls?

The prevalence of right and left IHI (partial, total, IHI subcriteria score) did not significantly differ between MDD patients and controls (Table 1). The IHI total score did not significantly differ between MDD patients and controls (patients: m(sd) = 3.5 (2.6), controls: m(sd) = 3.3 (2.6), t = 0.32, p = 0.74).

### 3.3 Is IHI related to specific sociodemographic or clinical characteristics in patients with MDD?

IHI (partial + total) was not significantly associated with age, sex, recurrent MDD, MDD duration, HDRS score at baseline and previous lifetime duration of antidepressant treatment (Table 2).

### 3.4 Is IHI related to hippocampal volume in patients with MDD?

HV did not differ between patients with or without IHI (partial + total) (Table 2).

### 3.5 Does hippocampal volume alone predict response/remission after antidepressant treatment in MDD patients?

Right hippocampal volumes were associated neither with sociodemographic characteristics (age: r = 0.05, p = 0.70; Sex: w = 470, p = 0.24) nor with clinical characteristics (recurrent MDD: w = 315, p = 0.67; MDD duration: r = 0.02, p = 0.90; HDRS score at baseline: r = 0.07, p = 0.60; antidepressant naive: w = 358; p = 0.30; previous antidepressant treatment duration: r = − 0.05; p = 0.74).

Left hippocampal volumes were associated neither with sociodemographic characteristics (age: r = 0.01, p = 0.97; Sex: w = 462, p = 0.97) nor with clinical characteristics (recurrent MDD: w = 291, p = 0.37; MDD duration: r = 0.10, p = 0.46; HDRS score at baseline: r = 0.08, p = 0.56; antidepressant naive: w = 310; p = 0.36; previous antidepressant treatment duration: r = − 0.07; p = 0.64).

HV (right and left) were not significantly associated with response and remission rates after antidepressant treatment (Table 3).

### 3.6 Does IHI alone predict response/remission after antidepressant treatment in MDD patients?

IHI (either left or right, partial + total IHI, Total IHI and IHI score ≥ 4) was not associated with remission, response, or HDRS score improvement, neither after one, three or six months post-treatment (Table 2**)**.

### 3.7 Does taking into account IHI improve the value of hippocampal volume in the prediction of response/remission after antidepressant treatment in MDD patients?

Table 3 shows the association between HV and antidepressant efficacy in the whole sample, and in patients with or without IHI.

Significant statistical interactions between left HV and left IHI were found to predict antidepressant remission after three months (p = 0.04) and six months (p = 0.03) of antidepressant treatment, these interactions remaining significant after adjustment on brain volume, age and gender (p = 0.04 and p = 0.04 respectively) and MRI acquisition sequences (p = 0.04 and p = 0.06 respectively). Accordingly, significant statistical interactions were found for response (p = 0.04, p = 0.07 after adjustment on brain volume, age and gender, and p = 0.03 after adjustment on MRI acquisition sequences) and HDRS improvement (p = 0.02, p = 0.04 after adjustment on brain volume, age and gender, and p = 0.004 after adjustment on MRI acquisition sequences) after six months of antidepressant treatment. Of note, no significant statistical interactions between right HV and right IHI were found for the prediction of antidepressant response/remission.

Thus, in patients without left IHI, HV at baseline did predict response, remission and HDRS score decrease post-treatment and remaining significant after adjustment on MRI acquisition sequences for HDRS score decreased after six month of antidepressant treatment (p = 0.01). On the contrary, in patients with left IHI, HV at baseline did not predict remission, response or HDRS score decrease post-treatment. Indeed, non-remitters and non-responders six months post-treatment had significantly smaller left hippocampus at baseline than remitters and responders respectively. A trend was also shown for three-month remission.

Since there are more patients in the subgroup of patients without IHI criteria, resulting in different powers that could explain the results, a case-control design on matched patients (15 with left IHI and 15 without left HI matched for age and sex) was secondary applied, confirming the statistical interaction of IHI and HV for HDRS score improvement six months post-treatment (p = 0.046) and at a trend for remission after three months (p = 0.06) and six months (p = 0.07) of treatment. This case-control design confirmed that, in patients without left IHI, HV at baseline was smaller for non-responders as compared to responders after six months of antidepressant treatment (1.97(0.34) cm^3^ vs 2.93(0.45) cm^3^, p = 0.02). There was a similar trend for remission after six months of antidepressant treatment (2.15(0.49) cm^3^ vs 2.94(0.52) cm^3^, p = 0.06). Accordingly, there was a significant correlation between HV at baseline and HDRS score decrease after 6 months of antidepressant treatment (r = 0.70, p = 0.04).

**Fig. S1:**
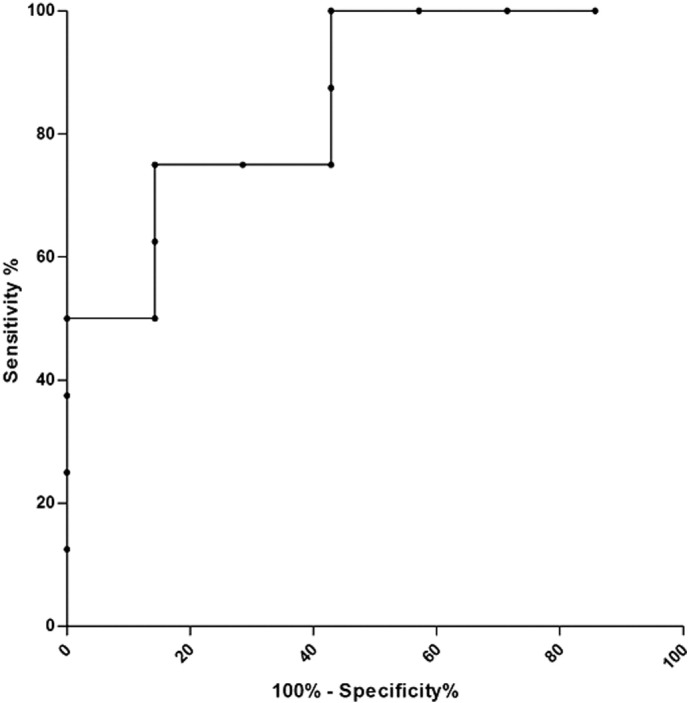
ROC curve for left hippocampal volume to predicts 6 months antidepressant remission in depressed patients without IHI.

To predict six-month remission with left HV in patients without IHI, a ROC analysis showed that the Area Under the Curve (AUC) was 0.86 (95%CI [0.66; 1.00]) (Fig. S1). In this sample, a left hippocampal volume > 2.50 cm^3^ had a 86% sensitivity, a 75% specificity, a 75% positive predictive value, and a 86% negative predictive value. A left hippocampal volume < 2.87 cm^3^ had a 57% sensitivity, a 100% specificity, a 100% positive predictive value and a 73% negative predictive value.

## 4 Discussion

To the best of our knowledge, this is the first study of IHI in MDD patients. IHI does not appear to be significantly more frequent in MDD patients than in controls, is not associated with MDD characteristics, is not predictive of treatment response when considered alone but is a confounder that decreases the relevance of hippocampal volume to predict remission after antidepressant treatment. Thus, taking into account IHI could improve the relevance of HV to predict antidepressant response or remission after antidepressant treatment.

Some clinical studies reported that HV could predict response or remission after antidepressant treatment of a Major Depressive Episode (MacQueen et al., 2008, Sheline et al., 2012). However, other studies failed to replicate these results (Frodl et al., 2008, Hsieh et al., 2002, Janssen et al., 2007, Vakili et al., 2000). Similarly to these previous negative reports, HV, when considered alone, did not significantly predict antidepressant response or remission in our sample, even if baseline HV were lower in patients with non-response/remission, but this difference was not statistically significant. This discrepancy between studies on the predictive value of HV could be due to the presence of IHI, which was not taken into account in previous volumetric studies. Indeed, in our study, while neither HV, nor IHI were predictive when considered alone, the interaction between HV and IHI predicted response and remission. Furthermore, a previous meta-analysis (Fu et al., 2013) reported that only right hippocampal volume and not left hippocampal volume predict antidepressant response/remission.

Our results about the prevalence of IHI in controls are similar to those of a recent study a community-based sample of over 2000 normal and young subjects in general population (Cury et al., 2015) for right and left IHI (total + partial right IHI frequency 16.7% vs 15.5% and total + partial left IHI frequency 33.3% vs 29%). In our study, the frequency of IHI was not significantly higher in MDD patients than in controls. This point does not argue for IHI as a neurodevelopmental risk marker for MDD. Nevertheless, this would need to be confirmed in a larger sample. Since the correlations between hippocampal volumes and HRDS improvement seem to be negative in patients with left IHI (although not significant) and positive in patients without IHI, this point should be further studied in greater sample sizes, in order to test whether or not these associations can reach statistical significance with greater statistical power.

Our study has the following limitations.

The first limitation is the statistical power of this study. While IHI was not significantly more frequent in MDD patients than in controls, our sample was relatively small and could have a too low statistical power to detect such a small effect. Indeed, the number of patients with three and six-month prospective assessments was relatively small, due to attrition. This is however similar to the attrition rate of other naturalistic studies in MDD such as STAR*D (Trivedi et al., 2006). Thus, there is a risk of false negative results (lack of power particularly in the subgroup of patients with IHI). Nevertheless, to control for this potential limitation, we performed a second analysis using a matched case-control design (with and without left IHI), which confirmed our primary results. Moreover, we observed no statistical difference of HV between MDD patients and controls and were unable to replicate the result of the recent paper of the ENIGMA Major Depressive Disorder working group published by Schmaal et al., 2015. This is probably due to the fact that the effect size described in the literature is too small to be detected with the power of our study (Kempton et al., 2011, Schmaal et al., 2015). The lack of power of our study may also explain that multiple adjustments may lead to statistical trends rather than statistically significant results. Of note, we used no corrections for multiple testing. Consequently, in future replication studies, a larger sample size and corrections for multiple comparisons would be required to confirm our preliminary exploratory results.

The second limitation of this study is that it relies only on automatic segmentation. It has been previously shown that the accuracy of automatic hippocampal segmentation is affected by the presence of IHI (Kim et al., 2012). It is thus possible that the confounding effect of IHI on HV could be due to a lower accuracy of the segmentation in IHI patients. Nevertheless, in our study, we performed systematic visual quality control of the segmentation results and only high quality segmentations were included in the analysis (three patients and four controls were excluded from the volumetric analyses). Furthermore, we did not find any statistically significant difference between patients with or without left IHI for segmentation quality scores (m(sd) 2.7(0.5) vs m(sd) 2.8(0.4), p = 0.20). However, adding measurements of a manual segmentation in addition to the automated one would have strengthened our results, and would be useful in future replication studies.

## 5 Conclusion

In conclusion, IHI is a confounder that decreases the predictive value of hippocampal volume to predict remission after antidepressant treatment.

An independent and large cohort using both automatic and manual segmentation is needed to replicate our results. Nevertheless, our results clearly demonstrate that IHI assessment is important when conducting volumetric studies. We recommend to systematically assess IHI in future research studies assessing hippocampal volume in MDD. Whether this assessment should be implemented in clinical practice has to be clarified by further studies.

The following is the supplementary data related to this article.

## Figures and Tables

**Table 1:** IHI frequency in patients and controls.

	Right hippocampus				Left hippocampus			
	Patients	Controls	Test	p	Patients	Controls	Test	p
Partial + total IHI n (%)	14 (23.3)	10 (16.7)	X^2^ = 0.8	0.36	23 (38.3)	20 (33.3)	X^2^ = 0.3	0.57
Total IHI n (%)	6 (10.0)	4 (6.7)	X^2^ = 0.4	0.51	13 (21.7)	9 (15.0)	X^2^ = 0.9	0.35
Partial IHI n (%)	8 (13.3)	6 (10.0)	X^2^ = 0.3	0.56	10 (16.4)	11 (18.3)	X^2^ = 0.1	0.81
C1 m(sd)	0.4 (0.5)	0.3 (0.4)	t = − 1.2	0.25	0.5 (0.5)	0.5 (0.5)	t = 0.1	0.93
C2 m(sd)	0.5 (0.5)	0.5 (0.5)	t = − 0.1	0.92	0.6 (0.6)	0.5 (0.5)	t = − 1.0	0.34
C3 m(sd)	0.4 (0.4)	0.4 (0.4)	t = − 0.5	0.65	0.6 (0.5)	0.6 (0.5)	t = 0.2	0.86
C4 m(sd)	0.0 (0.0)	0.0 (0.0)	na	na	0.0 (0.0)	0.0 (0.0)	na	na
C5 m(sd)	0.1 (0.5)	0.1 (0.5)	t = 0.0	1	0.5 (0.8)	0.4 (0.8)	t = − 0.2	0.82
IHI score m(sd)	1.3 (1.3)	1.3 (1.3)	t = − 0.1	0.89	2.2 (1.7)	2.0 (1.7)	t = − 0.4	0.70
IHI score ≥ 4 n (%)	6 (10)	6 (10.0)	t = 0.0	1	14 (23.3)	10 (16.7)	t = 2.0	0.15

Legends: MDD: Major Depressive Disorder; Major Depressive Episode; IHI: Incomplete Hippocampal Inversion; C: criteria.

X^2^ = Chi square**;** t = Student *t*-test.

**Table 2:** IHI and sociodemographical characteristics, clinical characteristics and antidepressant efficacy.

	Right IHI (partial + total)				Left IHI (partial + total)			
	Yes (n = 14)	No (n = 46)	Test	p	Yes (n = 23)	No (n = 37)	Test	p
Hippocampal volume (m(sd)) cm^3^	2.442 (0.602)	2.568 (0.617)	w = 264	0.40	2.577 (0.557)	2.494 (0.537)	w = 319	0.72
Age (m(sd))	46.4 (12.5)	45.5 (13.0)	w = 317.5	0.94	45.3 (11.3)	46.1 (13.8)	w = 457	0.64
Women (%)	64.3	58.7	X^2^ = 0.1	0.71	56.5	62.2	X^2^ = 0.2	0.67
Recurrent MDD (%)	64.3	69.6	X^2^ = 0.1	0.71	73.9	64.9	X^2^ = 0.5	0.46
MDD duration (m(sd)) years	11.6 (15.5)	7.7 (10.1)	w = 290	0.66	11.7 (14.3)	6 0.7 (9.1)	w = 327	0.17
HDRS (m(sd))	22.0	24.6	w = 412	0.12	24.0 (4.3)	24.0 (5.4)	w = 403	0.74
AD naïve (%)	7.1	28.3	X^2^ = 2.7	0.11	13.0	29.7	X^2^ = 2.2	0.14
Anterior AD duration (m(sd)) years	4.1 (5.6)	2.1 (4.1)	w = 198.5	0.09	3.5 (6.1)	1.9 (3.1)	w = 292.5	0.22
Non treated MDD duration (m(sd)) years	8.5 (12.7)	5.2 (8.9)	w = 252	0.41	7.2 (11.9)	5.2 (9.6)	w = 310.5	0.27
Antidepressant								
SSRI (%)	32.6	28.6	X^2^ = 0.4	0.82	30.4	32.4	X^2^ = 2.3	0.32
SNRI (%)	58.7	58.7			52.2	62.2		
TCA (%)	57.1	8.7			17.4	5.4		
M1	n = 13	n = 37			n = 18	n = 32		
% HDRS improvement (m(sd))	35.4 (29.7)	49.2 (26.0)	w = 306	0.15	44.8 (30.7)	46.1 (25.8)	w = 287.5	1
Response (%)	46.2	51.4	X^2^ = 0.1	0.74	61.1	43.8	X^2^ = 1.4	0.23
Remission (%)	15.4	27.0	X^2^ = 0.7	0.40	27.8	21.9	X^2^ = 0.2	0.63
M3	n = 9	n = 23			n = 11	n = 21		
% HDRS improvement (m(sd))	42.9 (32.3)	49.0 (32.5)	w = 113	0.71	39.7 (30.1)	51.3 (33.1)	w = 141	0.32
Response (%)	33.3	56.5	X^2^ = 1.4	0.24	36.4	57.1	X^2^ = 1.2	0.26
Remission (%)	33.3	26.1	X^2^ = 0.2	0.68	27.3	28.6	X^2^ = 0.0	0.94
M6	n = 7	n = 17			n = 8	n = 16		
% HDRS improvement (m(sd))	46.6 (45.7)	47.7 (38.6)	w = 58	0.95	40.2 (40.6)	51.0 (40.2)	w = 76	0.49
Response (%)	47.1	71.4	X^2^ = 1.19	0.28	56.2	50.0	X^2^ = 0.1	0.77
Remission (%)	42.9	41.2	X^2^ = 0.0	0.93	62.5	43.8	X^2^ = 0.1	0.77

Legends: IHI: Incomplete Hippocampal Inversion; MDD: Major Depressive Disorder; HDRS: Hamilton Depression Rating Scale; AD: antidepressant; SSRI: Selective Serotonin Reuptake Inhibitor; SNRI: Serotonin and Noradrenaline Reuptake Inhibitor; TCA: Imipraminics; M1: 1 month post-treatment, M3: 3 months post-treatment; M6: 6 months post-treatment; X^2^: Chi square; w: Wilcoxon test.

**Table 3:** Hippocampal volume as predictor of antidepressant efficacy with or without taking into account IHI.

	% HAMD-17 improvement	p	Responders	Non Responders	p	Remitters	Non Remitters	p
**Total sample (**n **=** **60)**								
M1 (n = 46)								
Right Hippocampus volume (m(sd)) cm^3^	r = − 0.07	0.64	2.63 (0.66)	2.53 (0.66)	0.97	2.76 (0.57)	2.52 (0.60)	0.45
Left hippocampus volume (m(sd)) cm^3^	r = 0.01	0.95	2.56 (0.60)	2.56 (0.57)	0.80	2.68 (0.61)	2.52 (0.57)	0.52
M3 (n = 30)								
Right Hippocampus volume (m(sd)) cm^3^	r = 0.08	0.67	2.70 (0.65)	2.50 (0.52)	0.35	2.86 (0.57)	2.49 (0.59)	0.14
Left hippocampus volume (m(sd)) cm^3^	r = 0.21	0.27	2.69 (0.71)	2.30 (0.40)	0.11	2.71 (0.73)	2.41 (0.54)	0.23
M6 (n = 24)								
Right Hippocampus volume (m(sd)) cm^3^	r = 0.09	0.64	2.72 (0.58)	2.54 (0.69)	0.42	2.75 (0.60)	2.54 (0.66)	0.42
Left hippocampus volume (m(sd)) cm^3^	r = 0.19	0.37	2.61 (0.72)	2.37 (0.55)	0.34	2.41 (0.54)	2.39 (0.53)	0.34

**Patients with left IHI (**n **=** **23)**								
M1 (n = 15)								
Left hippocampus volume (m(sd)) cm^3^	r = 0.03	0.91	2.55 (0.83)	2.73 (0.39)	0.52	2.55 (0.83)	2.59 (0.53)	0.99
M3 (n = 9)								
Left hippocampus volume (m(sd)) cm^3^	r = − 0.42	0.27	2.39 (0.96)	2.41 (0.34)	0.90	1.98 (0.62)	2.61 (0.58)	0.26
M6 (n = 7)								
Left hippocampus volume (m(sd)) cm^3^	r = − 0.57	0.20	1.98 (0.623)	2.65 (0.66)	0.40	1.98 (0.62)	2.64 (0.66)	0.40

**Patients without left IHI (**n **=** **37)**								
M1 (n = 29)								
Left hippocampus volume (m(sd)) cm^3^	r = 0.02	0.94	2.60 (0.552)	2.51 (0.56)	0.91	2.77 (0.48)	2.50 (0.56)	0.38
M3 (n = 19)								
Left hippocampus volume (m(sd)) cm^3^	r = 0.34	0.16	2.74 (0.635)	2.33 (0.38)	0.18	**2.95 (0.57)**	**2.39 (0.49)**	**0.09**
M6 (n = 15)								
Left hippocampus volume (m(sd)) cm^3^	r **=** **0.63**	**0.01**	**2.86 (0.54)**	**2.18 (0.42)**	**0.03**	**2.97 (0.53)**	**2.26 (0.43)**	**0.02**

Legends: IHI: Incomplete Hippocampal Inversion; M1: 1 month post-treatment; M3: 3 months post-treatment; M6: 6 months post-treatment.

## References

[bb0005] Andrade D.M., Krings T., Chow E.W., Kiehl T.R., Bassett A.S. (2013). Hippocampal malrotation is associated with chromosome 22q11.2 microdeletion. Can. J. Neurol. Sci..

[bb0010] Ansorge M.S., Hen R., Gingrich J.A. (2007). Neurodevelopmental origins of depressive disorders. Curr. Opin. Pharmacol..

[bb0015] Atlas S.W., Zimmerman R.A., Bilaniuk L.T., Rorke L., Hackney D.B., Goldberg H.I., Grossman R.I. (1986). Corpus callosum and limbic system: neuroanatomic MR evaluation of developmental anomalies. Radiology.

[bb0020] Bajic D., Wang C., Kumlien E., Mattsson P., Lundberg S., Eeg-Olofsson O., Raininko R. (2008). Incomplete inversion of the hippocampus–a common developmental anomaly. Eur. Radiol..

[bb0025] Bajic D., Kumlien E., Mattsson P., Lundberg S., Wang C., Raininko R. (2009). Incomplete hippocampal inversion-is there a relation to epilepsy?. Eur. Radiol..

[bb0030] Baker K., Chaddock C.A., Baldeweg T., Skuse D. (2011). Neuroanatomy in adolescents and young adults with 22q11 deletion syndrome: comparison to an IQ-matched group. NeuroImage.

[bb0035] Baulac M., De Grissac N., Hasboun D., Oppenheim C., Adam C., Arzimanoglou A., Semah F., Lehericy S., Clemenceau S., Berger B. (1998). Hippocampal developmental changes in patients with partial epilepsy: magnetic resonance imaging and clinical aspects. Ann. Neurol..

[bb0040] Bergouignan L., Chupin M., Czechowska Y., Kinkingnehun S., Lemogne C., Le Bastard G., Lepage M., Garnero L., Colliot O., Fossati P. (2009). Can voxel based morphometry, manual segmentation and automated segmentation equally detect hippocampal volume differences in acute depression?. NeuroImage.

[bb0045] Bernasconi N., Kinay D., Andermann F., Antel S., Bernasconi A. (2005). Analysis of shape and positioning of the hippocampal formation: an MRI study in patients with partial epilepsy and healthy controls. Brain.

[bb0050] Boronat S., Mehan W.A., Shaaya E.A., Thibert R.L., Caruso P. (2015). Hippocampal abnormalities in magnetic resonance imaging (MRI) of 15q duplication syndromes. J. Child Neurol..

[bb0055] Bronen R.A., Cheung G. (1991). MRI of the normal hippocampus. Magn. Reson. Imaging.

[bb0060] Campbell L.E., Daly E., Toal F., Stevens A., Azuma R., Catani M., Ng V., van Amelsvoort T., Chitnis X., Cutter W., Murphy D.G., Murphy K.C. (2006). Brain and behaviour in children with 22q11.2 deletion syndrome: a volumetric and voxel-based morphometry MRI study. Brain.

[bb0065] Chupin M., Hammers A., Bardinet E., Colliot O., Liu R.S., Duncan J.S., Garnero L., Lemieux L. (2007). Fully automatic segmentation of the hippocampus and the amygdala from MRI using hybrid prior knowledge. Med. Image Comput. Assist. Interv..

[bb0070] Chupin M., Hammers A., Liu R.S., Colliot O., Burdett J., Bardinet E., Duncan J.S., Garnero L., Lemieux L. (2009). Automatic segmentation of the hippocampus and the amygdala driven by hybrid constraints: method and validation. NeuroImage.

[bb0075] Colle R., Dupong I., Colliot O., Deflesselle E., Hardy P., Falissard B., Ducreux D., Chupin M., Corruble E. (2016). Smaller Hippocampal Volumes Predict Lower Antidepressant Response/remission Rates in Depressed Patients: A Meta-analysis.

[bb0080] Colliot O., Chetelat G., Chupin M., Desgranges B., Magnin B., Benali H., Dubois B., Garnero L., Eustache F., Lehericy S. (2008). Discrimination between Alzheimer disease, mild cognitive impairment, and normal aging by using automated segmentation of the hippocampus. Radiology.

[bb0085] Cury C., Toro R., Cohen F., Fischer C., Mhaya A., Samper-Gonzalez J., Hasboun D., Mangin J.F., Banaschewski T., Bokde A.L., Bromberg U., Buechel C., Cattrell A., Conrod P., Flor H., Gallinat J., Garavan H., Gowland P., Heinz A., Ittermann B., Lemaitre H., Martinot J.L., Nees F., Paillere Martinot M.L., Orfanos D.P., Paus T., Poustka L., Smolka M.N., Walter H., Whelan R., Frouin V., Schumann G., Glaunes J.A., Colliot O., Consortium I. (2015). Incomplete hippocampal inversion: a comprehensive MRI study of over 2000 subjects. Front. Neuroanat..

[bb0090] Dill V., Franco A.R., Pinho M.S. (2015). Automated methods for hippocampus segmentation: the evolution and a review of the state of the art. Neuroinformatics.

[bb0095] Frodl T., Jager M., Smajstrlova I., Born C., Bottlender R., Palladino T., Reiser M., Moller H.J., Meisenzahl E.M. (2008). Effect of hippocampal and amygdala volumes on clinical outcomes in major depression: a 3-year prospective magnetic resonance imaging study. J. Psychiatry Neurosci..

[bb0100] Fu C.H., Steiner H., Costafreda S.G. (2013). Predictive neural biomarkers of clinical response in depression: a meta-analysis of functional and structural neuroimaging studies of pharmacological and psychological therapies. Neurobiol. Dis..

[bb0105] Grosso S., Farnetani M.A., Berardi R., Bartalini G., Carpentieri M., Galluzzi P., Mostardini R., Morgese G., Balestri P. (2003). Medial temporal lobe dysgenesis in Muenke syndrome and hypochondroplasia. Am. J. Med. Genet. A.

[bb0110] Hamilton M. (1960). A rating scale for depression. J. Neurol. Neurosurg. Psychiatry.

[bb0115] Hsieh M.H., McQuoid D.R., Levy R.M., Payne M.E., MacFall J.R., Steffens D.C. (2002). Hippocampal volume and antidepressant response in geriatric depression. Int J Geriatr Psychiatry.

[bb0120] Janssen J., Hulshoff Pol H.E., Schnack H.G., Kok R.M., Lampe I.K., de Leeuw F.E., Kahn R.S., Heeren T.J. (2007). Cerebral volume measurements and subcortical white matter lesions and short-term treatment response in late life depression. Int. J. Geriatr. Psychiatry.

[bb0125] Kempton M.J., Salvador Z., Munafo M.R., Geddes J.R., Simmons A., Frangou S., Williams S.C. (2011). Structural neuroimaging studies in major depressive disorder. Meta-analysis and comparison with bipolar disorder. Arch. Gen. Psychiatry.

[bb0130] Kim H., Chupin M., Colliot O., Bernhardt B.C., Bernasconi N., Bernasconi A. (2012). Automatic hippocampal segmentation in temporal lobe epilepsy: impact of developmental abnormalities. NeuroImage.

[bb0135] Kupfer D.J., Frank E., Phillips M.L. (2012). Major depressive disorder: new clinical, neurobiological, and treatment perspectives. Lancet.

[bb0140] Lehericy S., Dormont D., Semah F., Clemenceau S., Granat O., Marsault C., Baulac M. (1995). Developmental abnormalities of the medial temporal lobe in patients with temporal lobe epilepsy. AJNR Am. J. Neuroradiol..

[bb0145] MacQueen G., Frodl T. (2011). The hippocampus in major depression: evidence for the convergence of the bench and bedside in psychiatric research?. Mol. Psychiatry.

[bb0150] MacQueen G.M., Yucel K., Taylor V.H., Macdonald K., Joffe R. (2008). Posterior hippocampal volumes are associated with remission rates in patients with major depressive disorder. Biol. Psychiatry.

[bb0155] Raininko R., Bajic D. (2010). “Hippocampal malrotation”: no real malrotation and not rare. AJNR Am. J. Neuroradiol..

[bb0160] Righini A., Zirpoli S., Parazzini C., Bianchini E., Scifo P., Sala C., Triulzi F. (2006). Hippocampal infolding angle changes during brain development assessed by prenatal MR imaging. AJNR Am. J. Neuroradiol..

[bb0165] Schmaal L., Veltman D.J., van Erp T.G., Samann P.G., Frodl T., Jahanshad N., Loehrer E., Tiemeier H., Hofman A., Niessen W.J., Vernooij M.W., Ikram M.A., Wittfeld K., Grabe H.J., Block A., Hegenscheid K., Volzke H., Hoehn D., Czisch M., Lagopoulos J., Hatton S.N., Hickie I.B., Goya-Maldonado R., Kramer B., Gruber O., Couvy-Duchesne B., Renteria M.E., Strike L.T., Mills N.T., de Zubicaray G.I., McMahon K.L., Medland S.E., Martin N.G., Gillespie N.A., Wright M.J., Hall G.B., MacQueen G.M., Frey E.M., Carballedo A., van Velzen L.S., van Tol M.J., van der Wee N.J., Veer I.M., Walter H., Schnell K., Schramm E., Normann C., Schoepf D., Konrad C., Zurowski B., Nickson T., McIntosh A.M., Papmeyer M., Whalley H.C., Sussmann J.E., Godlewska B.R., Cowen P.J., Fischer F.H., Rose M., Penninx B.W., Thompson P.M., Hibar D.P. (2015). Subcortical brain alterations in major depressive disorder: findings from the ENIGMA major depressive disorder working group. Mol. Psychiatry.

[bb0170] Sheline Y.I., Disabato B.M., Hranilovich J., Morris C., D'Angelo G., Pieper C., Toffanin T., Taylor W.D., MacFall J.R., Wilkins C., Barch D.M., Welsh-Bohmer K.A., Steffens D.C., Krishnan R.R., Doraiswamy P.M. (2012). Treatment course with antidepressant therapy in late-life depression. Am. J. Psychiatry.

[bb0175] Trivedi M.H., Rush A.J., Wisniewski S.R., Nierenberg A.A., Warden D., Ritz L., Norquist G., Howland R.H., Lebowitz B., McGrath P.J., Shores-Wilson K., Biggs M.M., Balasubramani G.K., Fava M., Team S.D.S. (2006). Evaluation of outcomes with citalopram for depression using measurement-based care in STAR*D: implications for clinical practice. Am. J. Psychiatry.

[bb0180] Vakili K., Pillay S.S., Lafer B., Fava M., Renshaw P.F., Bonello-Cintron C.M., Yurgelun-Todd D.A. (2000). Hippocampal volume in primary unipolar major depression: a magnetic resonance imaging study. Biol. Psychiatry.

